# Molecular Characterization of *Prunus lusitanica* L. Fruit Extracts and Their Health-Promoting Potential in Inflammation, Diabetes, and Neurodegenerative Diseases

**DOI:** 10.3390/ijms24108830

**Published:** 2023-05-16

**Authors:** Ana Abraão, Carlos Martins-Gomes, Raúl Domínguez-Perles, Ana Barros, Amélia M. Silva

**Affiliations:** 1 Centre for Research and Technology of Agro-Environmental and Biological Sciences (CITAB), University of Trás-os-Montes and Alto Douro (UTAD), 5001-801 Vila Real, Portugal; anasantosa1@hotmail.com (A.A.); camgomes@utad.pt (C.M.-G.); abarros@utad.pt (A.B.); 2 Phytochemistry and Healthy Foods Lab (LabFAS), Department of Food Science and Technology (CEBAS-CSIC), University Campus of Espinardo, Edif. 25, 30100 Murcia, Spain; rdperles@cebas.csic.es; 3 Department of Biology and Environment (DeBA-ECVA), University of Trás-os-Montes and Alto Douro (UTAD), 5001-801 Vila Real, Portugal

**Keywords:** natural bioactive compounds, *Prunus lusitanica* L., antioxidant activity, neuroprotection, antiproliferative activity, anti-inflammatory activity

## Abstract

*Prunus lusitanica* L. is a shrub belonging to the genus *Prunus* L. (Rosaceae family) that produces small fruits with none known application. Thus, the aim of this study was to determine the phenolic profile and some health-promoting activities of hydroethanolic (HE) extracts obtained from *P. lusitanica* fruits, harvested from three different locations. Qualitative and quantitative analysis of extracts was performed using HPLC/DAD-ESI-MS and antioxidant activity was assessed by in vitro methods. Antiproliferative/cytotoxic activity was determined on Caco-2, HepG2, and RAW 264.7 cells, anti-inflammatory activity was assessed using lipopolysaccharide (LPS)-stimulated RAW 264.7 cells, and the antidiabetic, antiaging, and neurobiological action of extracts was determined in vitro by assessing their inhibitory effect against the activity of α-amylase, α-glucosidase, elastase, tyrosinase, and acetylcholinesterase (AChE). Results showed that *P. lusitanica* fruit HE extracts from the three different locations showed identical phytochemical profile and bioactivities, although small differences were observed regarding the quantities of some compounds. Extracts of *P. lusitanica* fruits contain high levels in total phenolic compounds, namely, hydroxycinnamic acids, as well as flavan-3-ols and anthocyanins, primarily cyanidin-3-(6-*trans*-*p*-coumaroyl)glucoside. *P. lusitanica* fruit extracts have a low cytotoxic/antiproliferative effect, with the lowest IC_50_ value obtained in HepG2 cells (352.6 ± 10.0 μg/mL, at 48 h exposure), but high anti-inflammatory activity (50–60% NO release inhibition, at 100 μg/mL extract) and neuroprotective potential (35–39% AChE inhibition, at 1 mg/mL), and moderate antiaging (9–15% tyrosinase inhibition, at 1 mg/mL) and antidiabetic (9–15% α-glucosidase inhibition, at 1 mg/mL) effects. The bioactive molecules present in the fruits of *P. lusitanica* deserve to be further explored for the development of new drugs of interest to the pharmaceutical and cosmetic industry.

## 1. Introduction

Due to the increase in life expectancy, as well as the boost in unhealthy lifestyles (e.g., sedentary habits and hypercaloric diets), the risk of metabolic diseases, such as type 2 diabetes mellitus, obesity, and hyperlipidemias, as well as of cancer and neurodegenerative diseases, is increasing [1]. In addition to the higher prevalence of metabolic diseases, the high cost of synthetic drugs used to treat their symptoms, as well as their adverse effects, influences the development of new synthetic pharmaceutical formulations and favors the discovery and screening of natural molecules [2,3], which usually present lower toxicity and cost. Other conditions are commonly associated with metabolic diseases, such as inflammatory processes and oxidative stress, resulting from imbalance in redox metabolism, which are also aggravated by the lifestyles mentioned above [4,5,6]. Thus, changes in lifestyle, such as ingestion of foods rich in antioxidants and in nutraceutical molecules, may prevent or treat a variety of these diseases or associated symptoms, as well as contribute to a healthier status. Natural products have long been studied as sources of molecules to target the inflammatory process, both chronical and acute, mediated by trauma or infection, through the modulation of signaling cascades and/or responses of immune system cells (e.g., macrophages and neutrophils) [7]. Regarding antioxidant activity, there is a growing effort to validate the results obtained by chemical assays in biological models, aiming to understand how to exploit the antioxidant potential of natural products to reduce oxidative stress in vivo. Furthermore, studies suggest that, as many metabolic diseases depend on several factors, the use of “polyherbal” formulations or combinations of drugs and herbs as a therapeutic approach may improve the treatment efficiency [2]. This may also apply to extracts, which contain various bioactive molecules, whose heterogeneity may be a key factor to address the multifactorial nature of metabolic diseases. Nevertheless, this requires intensive screening of molecular phytochemical compositions, as well as the study of their bioactivities.

Many plant species have been screened for their phytochemicals and respective bioactivities, resulting in the many natural products that are currently used as treatment drugs, or have served as inspiration to create more effective and less cytotoxic drugs [8]. Among these, galantamine (isolated from several members of the Amaryllidaceae family, such as *Leucojum* spp. and *Galanthus* spp.) used in Alzheimer’s disease treatment [9], taxol (originally isolated from *Taxus brevifolia* L.) used in chemotherapy [10], and artemisinin (isolated from *Artemisia annua* L.) used as an antimalarial [11] are some of the natural compounds in the list of drugs currently approved, a large portion of which consists of natural compounds or derivatives of natural compounds [8]. Another example is the antidiabetic drug metformin, whose development was based on the phytochemical galegine, present in *Galega officinalis* L., a plant historically used in traditional medicine to treat symptoms associated with diabetes [12,13]. Another prime example is acetylsalicylic acid, sold as aspirin, a nonsteroidal anti-inflammatory drug originally derived from salicin [14].

Natural products, such as plants and fruits, contain a diversity of biologically active molecules, which play a crucial role in regulating several cellular and extracellular processes, resulting in antioxidant, antiproliferative, anti-inflammatory, antidiabetic, and antiaging effects, among others [15,16,17]. Most of the biological effects result from direct interaction of phytochemicals with key enzymes involved in relevant metabolic processes. For example, compounds with ability to inhibit acetylcholinesterase (AChE) are promising molecules to develop new drug leads for neurodegenerative diseases treatment, such as Alzheimer’s and Parkinson’s diseases, as these pathologies are characterized by a decrease in acetylcholine release [9,18,19]. Another target enzyme is tyrosinase, whose involvement in Parkinson’s disease is still being unveiled; however, increased neuromelanin production in *substantia nigra* contributes to the degradation of dopaminergic neurons and is considered a hallmark of disease progression [20,21,22]. Furthermore, the search for anti-tyrosinase agents is also related to its role in skin melanin production, being a major target of cosmetic products, such as whitening agents [23]. Phytochemicals that inhibit α-amylase and α-glucosidase activity are sought as antidiabetic agents or hypoglycemic agents, as these enzymes are responsible for carbohydrate hydrolysis at the intestinal level; thus, their inhibition decreases or delays glucose absorption, thereby preventing postprandial hyperglycemia [24].

In this respect, screening new and unexplored plant species concerning their phytoconstituents and respective bioactivities is an ongoing process, aiming to identify and discover new potential drugs. Among the unexplored plants, *Prunus lusitanica* L., belonging to the *Prunus* L. genus of the Rosaceae family (Prunaceae or Amygdaloideae subfamily), is an example. This plant, considered a relict tree from the Tertiary, is distributed mainly in the Iberian Peninsula, Northern Morocco, and Macaronesia [25]; it is also known as laurel cherry, cherry bay, or Portuguese laurel [26]. *P. lusitanica* is an evergreen shrub with alternate and oval leaves, resembling those of bay laurel, whose small flowers (10–15 mm diameter) are produced on erect or spreading racemes in late spring, and whose fruits, consisting of small cherry-like drupes (8–13 mm in diameter), ripen in late summer or early autumn and are of dark purple or black color [27]. This species is mainly used as ornamental tree and easily adapts to its surrounding environment, contributing to a maintenance of ecological balance and ecosystem sustainability [26]. Despite its unique qualities and relative importance, only a few studies are available in the literature concerning *P. lusitanica*’s chemical composition. *P. lusitanica* leaf extracts are rich in triterpenoids, such as 2α,3α-dihydroxyurs-12-en-28-oic acid, ursolic acid, ursol aldehyde [28], and friedelin 1 [26]. Recently, our group reported the polyphenol composition of *P. lusitanica* fruit extracts, which consisted mainly of hydroxycinnamic acids, flavan-3-ols, anthocyanins, and flavonols [29]. The nutritional value of *P. lusitanica* fruit was recently reported, highlighting this fruit as rich in amino acids, both essential (e.g., arginine, leucine, and lysine) and non-essential, as well as minerals and fibers [22]. Considering the rich content of these fruits in phytonutrients, particularly phenolic compounds, and as this fruit is not commonly added to human diets, there is a need to further explore the potential health effects of this fruit, by performing an assessment of the bioactivities of *P. lusitanica* fruit extracts using in vitro models.

The aim of this study was to determine the phenolic profile and bioactivities of hydroethanolic extracts obtained from ripe *P. lusitanica* L. fruits, with different provenances. The antiproliferative activity of the extracts was determined in two human cancer cell lines, Caco-2 and HepG2, the anti-inflammatory activity was assessed in LPS-stimulated RAW 264.7 cells, and the potential antidiabetic, antiaging, and neurobiological actions of extracts were determined in vitro by assessing the extracts’ inhibitory effect against the activity of α-amylase, α-glucosidase, elastase, tyrosinase, and acetylcholinesterase (AChE). It should be emphasized that, to the best of our knowledge, this is the first report exploring the in vitro biological activity of *P. lusitanica* L. fruit extracts.

## 2. Results and Discussion

### 2.1. Extraction Yields, Phytochemical Characterization, and Antioxidant Activity

As shown in Table 1, the hydroethanolic extracts of *P. lusitanica* fruits, from the three different locations (see Section 3 for details), presented identical yields (54–59%, *w*/*w*). Although extracts from fruits harvested at PL2 showed, on average, higher extraction yield, this was not statistically different from those harvested at PL1. Yields in the same range have been obtained for hydroethanolic extracts of other fruits from the *Prunus* genus; for example, pulp extracts from *Prunus domestica* subsp. *domestica* and *P. domestica* subsp. *syriaca* yielded 63% and 46% [30] using the same ethanol/water ratio as this study (70:30).

Regarding the molecular composition of these extracts, HPLC-PAD–ESI-MS/MS (high-performance liquid chromatography with photodiode array detection coupled to electrospray ionization tandem mass spectrometry) analysis was used for the identification and quantification of the phenolic compounds present in *P. lusitanica* fruit extracts, and the results are shown in Table 2. According to a previous study [29], 28 phenolic compounds were identified and quantified in all *P. lusitanica* fruit extracts, regardless of the geographic location where the fruits were harvested (Table 2). These 28 compounds belonged to different classes, namely, hydroxycinnamic acids, flavonoids (e.g., flavan-3-ols, flavonols, anthocyanins), and secoiridoids, with hydroxycinnamic acids being the most represented both in number and in quantity. As observed, the phenolic profile of *P. lusitanica* fruit extracts from the different locations was identical with respect to the compounds present; however, some differences were observed when analyzing the quantities of the different compounds. Extracts of *P. lusitanica* fruits collected in Vila Real (PL1) had a higher amount of total phenolic compounds, which contributed to the higher amount of total hydroxycinnamic acids. However, this extract contained the lower amount of total flavan-3-ols and an intermediate level of total anthocyanins. On the other hand, samples from Mata da Margaraça (PL3) contained a higher amount of total flavan-3-ols (more than double that of the Vila Real sample) and a higher amount of total anthocyanins (more than double that of the Pardieiros PL2 sample). These results show that geographical location and, thus, local edaphoclimatic conditions affected the quantities of phenolic compounds produced by fruits. The biosynthesis of anthocyanins and anthocyanidins occurs most exclusively in plants, in a branch of the phenylpropanoid pathway, which is also involved in the biosynthesis of other flavonoids [31,32]. This biosynthetic pathway is one of the most studied regarding the production of plant secondary metabolites. The various enzymes involved in the different steps, as well as the factors that regulate their expression and activity, have also been well described in many publications (e.g., [31,32,33,34]). Several climatic factors have been reported to affect anthocyanin biosynthesis, e.g., light (intensity and type of radiation) and temperature [35]. Light intensity and ultraviolet radiation have been shown to stimulate anthocyanin production by inducing structural genes and regulatory factors involved in this biosynthetic pathway [35,36]. On the other hand, temperature seems to negatively influence the accumulation of anthocyanins, as cold weather favors their accumulation compared to warm weather, explained by the fact that higher temperatures inhibit structural genes [35]. Comparing the three locations, the average temperature was practically identical; however, nighttime temperatures in PL1 and PL3 could reach lower values than in PL2 [37] due to altitude and the microenvironment effect, partly explaining the lower values of anthocyanins in PL2 (Table 2). The PL1 and PL3 locations were also favored by sunlight exposure, which also contributed to the accumulation of anthocyanins. Hydroxycinnamic acids are also synthesized through the phenylpropanoid pathway [34]. Factors such as drought or senescence conditions were reported to increase hydroxycinnamic acid biosynthesis [38], due to the activation of structural genes. However, other factors, such as soil composition and microenvironment, might also influence the production of these secondary metabolites; these factors were not the focus of the current research.

It is interesting to note that, in extracts obtained from the fruits of other *Prunus* species, hydroxycinnamic acid and their derivatives are also the most abundant compounds, e.g., *P. domestica* subsp. *syriaca* [30] and *Prunus spinosa* (blackthorn) [39]. Several compounds were also identified as present in all samples, such as acetyl-*p*-coumaroylsucroses (peaks 3, 11, 12, 13–19, and 22–26, with peaks 11, 13, 19, and 26 present in higher amounts; Table 2), corroborating the pattern for this fruit [29]. Derivatives of acetyl-*p*-coumaroylsucrose were also reported to be present in fresh flowers of *Prunus mume* [40].

Concerning the antioxidant activity, three methods were performed, the ferric ion-reducing antioxidant power assay (FRAP) and the ABTS and DPPH free-radical-scavenging assays; the results are shown in Table 1. The same trend of antioxidant activity was observed in the different assays, with the antioxidant activity of samples ranked PL3 (Mata da Margaraça) > PL1 (Vila Real) > PL2 (Pardieiros). The antioxidant activity is related to the phytochemical profile, as well as the quantities of each class of compounds [17,29]. *P. lusitanica* fruit extracts collected in Mata da Margaraça showed the highest antioxidant activities, as assessed by the three different antioxidant assays, which can probably be attributed to the higher content of flavan-3-ols, e.g., B-type proanthocyanidin trimer (Table 2). The PL3 sample also contained a higher content of total anthocyanins (Table 2), which may have contributed to the high antioxidant activity (Table 1); this is corroborated by the fact that several extracts of the purple variety of plum, *P. domestica* subsp. *domestica*, showed higher antioxidant activity than the yellow variety, *P. domestica* subsp. *syriaca*, as the purple color is mainly due to the anthocyanin content [30].

The antioxidant potential is closely related to the molecular structure of polyphenols, which is influenced by the number and position of hydroxyl groups; thus, it is expected that different polyphenolic compositions produce different antioxidant activity, depending on the chemical structure of components, with flavan-3-ols and anthocyanins having higher antioxidant potential [17,41,42]. For the results presented herein, summing the total flavan-3-ols + total anthocyanins gave the same order (PL3 > PL1 > PL2) as that observed for the antioxidant activity, corroborating the structure-related antioxidant activity. Thus, in this case, antioxidant activity seemed to be directly correlated with the extracts’ content of total flavan-3-ols + total anthocyanins. Usually, phytochemical- and antioxidant-rich plant products have significant bioactive potential and are worth screening for new pharmaceutical and/or cosmeceutical applications.

### 2.2. Antiproliferative/Cytotoxic Activity of P. lusitanica Fruit Extracts

**Figure 1:**
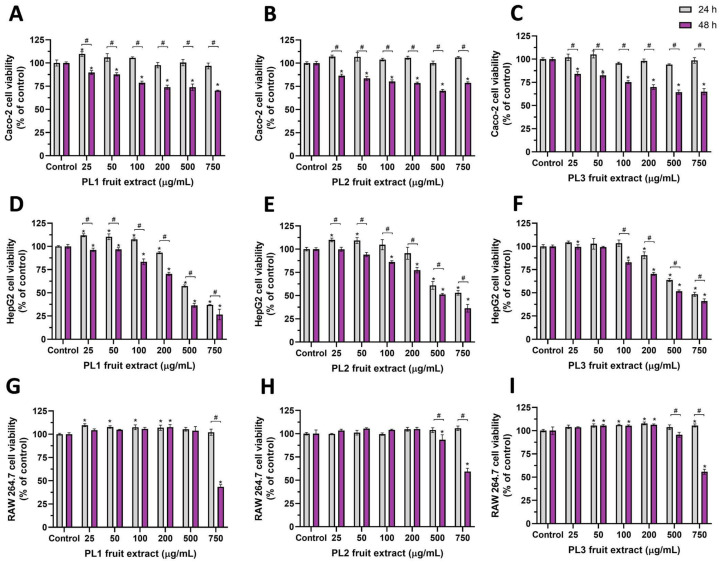
Antiproliferative/cytotoxic effect of *P. lusitanica* L. fruit extracts on Caco-2 (**A**–**C**), HepG2 (**D**–**F**), and Raw 264.7 (**G**–**I**) cells, for two exposure times: 24 h (gray bars) and 48 h (dark-pink bars). Results are expressed as the mean ± SD (n = 4). * Statistically significant difference (*p* < 0.05) between the control and sample concentrations at respective incubation times; ^#^ statistically significant difference (*p* < 0.05) between exposure periods at the same concentration.

The antiproliferative/cytotoxic activity of *P. lusitanica* fruit HE extracts was assessed using the Alamar Blue (AB) assay and three selected cell lines: the human cell lines Caco-2 and HepG2 as models of enterocyte and hepatocyte cells, respectively, and the murine macrophage cell line, RAW 264.7 cells. HE extracts of *P. lusitanica* fruits harvested in the three different locations (PL1, PL2, and PL3) were used. Cells were incubated, for 24 h or 48 h, with different concentrations of extracts (25, 50, 100, 200, 500, and 750 μg/mL) as depicted in Figure 1, and the results were compared with the respective positive control cells (nonexposed cells). As observed, 24 h exposure of Caco-2 cells with *P. lusitanica* fruit HE extracts did not produce a cytotoxic effect as cell viability was close to 100% of the control, at all tested concentrations (Figure 1A–C: gray bars), with no statistical difference (*p* > 0.05). However, exposure of Caco-2 cells to extracts for 48 h dose-dependently reduced cell viability, with the viability reduction profile being identical for the extracts obtained from the fruits harvested at the different locations (Figure 1A–C: purple bars). Despite the identical profile, cell viability after 48 h exposure to 750 μg/mL of LP1 or LP2 extract was about 70–78% of the control, reducing slightly (65% of the control) when exposed to 750 μg/mL of LP3 extract. The LP3 extract had higher amounts of flavan-3-ols and total anthocyanins (Table 2), which may have reduced the rate of proliferation of these cells, since a reduction in cell proliferation has been reported for several cell lines exposed to various flavan-3-ols [43,44] and anthocyanin-rich extracts [45].

As shown in Figure 1D–F, *P. lusitanica* fruit HE extracts dose-dependently reduced HepG2 cell viability with an identical profile at both exposure times but with slightly different potencies, with longer exposure being more cytotoxic. As observed, 24 h exposure to concentrations up to 100 μg/mL did not reduce HepG2 cell viability (cell viability: ~100% of the control or above), but 48 h exposure to the same concentrations reduced cell viability by about 15–20% (cell viability: 80–85% of the control). At the highest tested concentration of 750 μg/mL, cell viability was reduced to 50% of the control or even lower (Figure 1D–F). The IC_50_ values (Table 3) show that HepG2 cells were the most sensitive to these extracts; however, for the 24 h exposure, the IC_50_ values were higher than 500 μg/mL, denoting a very low cytotoxic effect. On the other hand, RAW 264.7 cells were the least affected (Figure 1G–I).

On the other hand, in Caco-2 and HepG2 cells, the obtained IC_50_ values were all higher than 350 μg/mL, which indicates that these extracts could be considered weakly active or inactive, with respect to antiproliferative/anticancer activity, according to the criteria of Geran et al. [46] (weakly active when IC_50_ 0.20–0.50 mg/mL and inactive when IC_50_ > 0.50 mg/mL). Identical IC_50_ values were reported for the anthocyanin-rich fractions of blueberry (IC_50_ ~500 μg/mL) and blackcurrant (IC_50_ ~300 μg/mL) juices, against A2780, HeLa, and B10F10 cancer lines [47].

As observed, for RAW 264.7 cells (Figure 1G–I), only the higher tested concentrations, for the 48 h exposure, reduced cell viability to values close to 50% of the control (Figure 1H,I) or below (Figure 1G). The calculated IC_50_ values were close to 750 μg/mL (PL1 extract, 48 h exposure) or higher (Table 3, Figure 1G–I). According to Geran et al. [46], these extracts could be considered inactive against RAW 264.7 cells, since IC_50_ > 0.50 mg/mL (Table 3). The viability of Caco-2, HepG2 RAW 264.7 cells was not affected by concentrations up to 500 μg/mL of anthocyanin-rich elderberry (*Sambucus nigra* L.) extracts [48].

### 2.3. Anti-Inflammatory Activity of Prunus lusitanica Fruit Extracts

**Figure 2:**
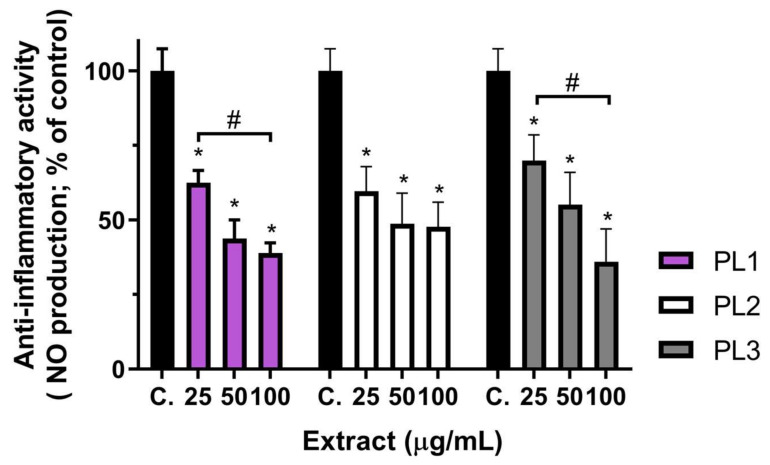
Anti-inflammatory activity of *P. lusitanica* L. fruit extracts expressed as the inhibition of nitric oxide (NO) release by LPS-stimulated RAW 264.7 cells. Results are expressed as the mean ± SD (*n* = 4 independent assays). * Statistical differences (*p* < 0.05) between samples and the positive control (LPS-stimulated cells not exposed to extracts); ^#^ statistical differences (*p* < 0.05) between concentrations of the same sample.

The anti-inflammatory activity of *P. lusitanica* fruit extracts (from the three locations) was evaluated on RAW 264.7 cells by their capacity to decrease the lipopolysaccharide (LPS)-induced nitric oxide (NO) release. The anti-inflammatory effect was evaluated at noncytotoxic concentrations, taking into account the results shown in Figure 1. RAW 264.7 cells were pre-exposed to *P. lusitanica* fruit extracts at concentrations up to 100 µg/mL, for 24 h, and then challenged with LPS (see Section 3 for details). Results concerning the anti-inflammatory potential of these extracts are shown in Figure 2. As observed, *P. lusitanica* fruit extracts from the different locations dose-dependently reduced NO release from LPS-stimulated RAW 264.7 cells, indicating that these extracts had the ability to target the TLR-4 (Toll-like receptor 4) pathway, as well as reduce iNOS (inducible nitric oxide synthase) expression and/or activity, resulting in lower amounts of released NO. PL1 and PL2 extracts at 25 µg/mL reduced NO release by about 40% (NO level: ~60% of the control), whereas PL3 reduced NO release to 70% of the control (Figure 2).

PL2 did not produce a significant dose-dependent inhibition of NO production at concentrations up to 100 µg/mL. On the other hand, PL1 and PL3 produced significant dose-dependent inhibition, inducing a significantly lower NO production at the highest tested concentration (100 µg/mL), with PL3 being the most effective extract, inducing ~64% inhibition (36% of the control’s NO production). PL3 presented a higher content of anthocyanins and flavan-3-ols (Table 2), which was likely correlated with the higher inhibition of NO release observed. Some of these compounds have been reported to produce anti-inflammatory activity.

Cyanidin-3-glucoside, for example, has been shown to reduce NO production in LPS-stimulated RAW 264.7 cells and in an intestinal cell model (HT-29), where it also induced a reduction in iNOS and cyclooxygenase 2 (COX-2) expression [49,50]. This activity was also observed for extracts obtained from other species rich in pro-anthocyanidin derivatives, which were present in a higher content in PL3 compared to PL1 and PL2. Some examples are extracts of cranberry [51], blueberry [52], elderberry [48], or grape [53]. Similarly, *Musa acuminata* extracts, rich in acetylated *p*-coumaroyl sucrose derivatives, such as various mumeoses and prunoses, also produced anti-inflammatory activity [54].

Specifically, in the *Prunus* genus, *Prunus armeniaca* leaf extracts were shown to inhibit COX-1 and COX-2 activity in in vitro assays [55]. Methanol and ethyl acetate extracts of *Prunus yedoensis* (50 and 100 µg/mL) reduced both NO and PGE_2_ (prostaglandin E2) production, as well as iNOS and COX-2 expression, in LPS-stimulated RAW 264.7 cells [56]. The anti-inflammatory effect of *Prunus avium* hydroethanolic extracts in RAW 264.7 cells was also evaluated, with leaf, stem, and flower extracts producing a dose-dependent inhibition of NO production, at concentrations ranging 50–400 µg/mL [57].

### 2.4. Assessment of the Inhibitory Effect of P. lusitanica Fruit Extracts on the Activity of Metabolically Relevant Enzymes

*P. lusitanica* fruit extracts were evaluated for their ability to inhibit key enzymes identified as therapeutic targets of common degenerative and metabolic pathologies, e.g., in the case of Alzheimer’s disease (acetylcholinesterase; AChE [58]), Parkinson’s disease (tyrosinase [59]), and diabetes mellitus (α-amylase and α-glucosidase [60,61]), as well as enzymes targeted for cosmetic purposes, such as skin-whitening (tyrosinase) and antiaging products (elastase) [23,62,63].

Two concentrations of *Prunus lusitanica* fruit HE extracts (from the three locations) were chosen: 0.5 and 1 mg/mL; the results are presented in Table 4.

Phytochemicals with the ability to inhibit AChE activity are promising molecules in the development of new drug leads for the treatment of neurodegenerative diseases, such as Alzheimer’s and Parkinson’s diseases, as these are characterized by decreased acetylcholine release [18]. As observed in Table 4, all samples dose-dependently inhibited AChE activity. The greatest inhibitory effect was observed for 1 mg/mL *P. lusitanica* extract, which inhibited AChE activity by 34% to 40% (Table 4). The PL1 extract produced the highest average inhibitory effect, at both 0.5 and 1 mg/mL, although the observed effect was not statistically significant at the latter concentration (*p* > 0.05) when compared with the other extracts. The highest inhibition induced by PL1 can likely be explained by the higher content of total phenolic compounds (Table 2), as this extract presented both the highest AChE inhibition and the greatest content of total phenolic compounds. PL3, the extract with a lower average AChE inhibition, presented the highest content of anthocyanins and flavan-3-ols, thus inferring that these components present a lower correlation with AChE inhibition. Among the main phytochemicals quantified (Table 2), various hydroxycinnamic acids and derivatives have been described as AChE inhibitors, e.g., *p*-coumaric, ferulic, and sinapic acids [64,65]. Regarding other *Prunus* spp., the hydroethanolic extracts of three cultivars of *Prunus armeniaca* L., containing various *p*-coumaroyl derivatives and flavanols, were shown to inhibit AChE activity, with IC_50_ values ranging between 5.53 and 10.33 mg/mL [55], concentrations ~5.5 and ~10 times higher than the highest concentration tested herein for *P. lusitanica* that presented ~40% inhibition (Table 4). Phenolic compounds present in sour cherry juice (*Prunus cerasus*), such as gallic or chlorogenic acids, showed inhibitory activity against AChE and monoamine oxidase A, with both enzymes being relevant in neuroprotective activity [66]. In another study, essential oils of *P. armeniaca* and *Prunus domestica* L. also inhibited AChE activity, with IC_50_ values ranging between 0.09 and 0.172 mg/mL [67]. These studies infer that *Prunus* spp. are sources of biologically active compounds with anti-AChE activity, which deserve further study.

Concerning the ability to inhibit tyrosinase activity, the same trend was observed as for AChE inhibition (Table 4), with PL1 extracts producing the greatest inhibitory effect (15% inhibition, at 1 mg/mL); the inhibition efficiency was in the order PL1 > PL2 > PL3, showing a dose-dependent effect (Table 4). This implies that these extracts could have some potential to counter neurodegeneration in Parkinson’s disease (by inhibiting neuromelanin production), as well as a cosmeceutical effect in skin-whitening agents [20,21,22]. Other *Prunus* species have been addressed for their potential as melanogenesis inhibitors. *Prunus serrulata* leaf aqueous and hydroethanolic extracts presented a higher potential as tyrosinase inhibitors [68] than *P. lusitanica* fruit extracts (Table 4), with 0.1 mg/mL aqueous and hydroethanolic extracts inhibiting tyrosinase activity by 63% and 39%, respectively [68]; however, the extract composition was different. A higher inhibitory effect against tyrosinase was also reported for hydroethanolic extracts of *Prunus persica* leaves that produced 14% inhibition at 0.5 mg/mL, when compared to *Prunus persica* fruit extracts that only produced 2% inhibition at the same concentration [69]. Thus, the tyrosinase-inhibitory effect of *P. persica* fruit extracts [69] is in line with the results here presented for *P. lusitanica* fruit extracts (Table 4). The ethanolic extracts from leaves of several varieties of *P. domestica* produced inhibition of tyrosinase activity, ranging from 0% to 11.60% at 3.43 mg/mL [70]. The data presented and discussed herein highlight the applicability of *Prunus* species to search for new cosmetic ingredients, as they combine antioxidant (Table 1) and tyrosinase-inhibitory activity, which might contribute to the regulation of melanin synthesis and attenuation of oxidative and age-related skin hyperpigmentation.

None of the extracts showed the ability to inhibit elastase activity, up to 1 mg/mL (Table 4). The absence of an elastase-inhibitory effect was also observed for methanolic extracts of elderberries (*Sambucus nigra* L.); these fruits were also rich in cinnamic acids and anthocyanins, such as cyanidin-3-*O*-glucoside [71], in line with the composition of *P. lusitanica* (Table 2), indicating that these compounds do not have ability to inhibit elastase. On the other hand, extracts obtained from leaves of several *Prunus* species (using organic solvents, such as hexane, ethyl acetate, and ethanol), were reported to produce anti-elastase activity depending on the solvent used (e.g., [72]). Stierlin et al. [72] obtained different extracts from leaves of various *P. domestica* varieties, showing that, although hexane and ethyl acetate extracts produced anti-elastase activity, the ethanolic extract produced no inhibition at 3.43 mg/mL. However, using a different extraction method, with ethanol as the solvent, Plainfossé et al. [70] reported significant anti-elastase activity for leaves of *P. domestica.* This indicates that both the solvent and the extraction method interfere with the extract’s bioactivities, which might be directly correlated with its phytochemical composition. Extracts from other sources have shown promising results concerning anti-elastase activity, such as extracts obtained from stems of different grape (*Vitis vinifera* L.) varieties, which inhibited 60% to 98% of elastase activity at 1 mg/mL [73]. Moreover, hydroethanolic extracts of orange thyme inhibited about 50% of elastase activity at 0.5 mg/mL [74]. Thus, although *Prunus* spp. extracts may present antiaging potential, the use of solvents with higher polarity is necessary to extract phytochemicals that contribute to this activity, which cannot be extracted using ethanol, as shown above (Table 4).

Concerning antidiabetic potential, unlike anti-AChE and anti-tyrosinase activity, no significant dose-dependent inhibition was observed for anti-α-amylase activity, with the exception of PL1 (Table 4), which also produced the highest average inhibition at 1 mg/mL. As observed, extracts produced low α-amylase-inhibitory activity. However, antidiabetic activity at the enzymatic level may also be exerted as α-glucosidase inhibition. Both PL1 and PL2 produced the highest α-glucosidase inhibition (~15%; Table 4); although, on average, the inhibitory effect increased with concentration, the differences were not statistically significant (*p* > 0.05). Despite the LP2 extract presenting a lower content of total phenolic compounds (Table 2), the unique phytochemical profile of each extract and the synergisms within its phytochemicals may have contributed to the variations observed. The LP3 extract produced a lower α-glucosidase-inhibitory effect; indeed, this extract contained lower amounts of di- and triacetylated *p*-coumaroyl derivatives, in the glycosylated form, which can promote competitive inhibition; however, further studies are needed to solidify this conclusion. Various individual components such as hydroxycinnamic acids [75], isoquercetin, rutin [76], and cyanidin-3-glucoside [77] have been shown to inhibit α-glucosidase; thus, these molecules were expected to contribute to the inhibition observed herein as they were identified in the extracts (Table 2). Regarding studies in other *Prunus* spp., Nan et al. [78] reported, in *Prunus mume* flower bud hydromethanolic extracts, the direct binding of phenolic compounds to the enzyme, contributing to the inhibition of α-glucosidase binding activity evaluated using an online methodology. Among the compounds studied by Nan et al. [78] in *Prunus mume* extracts (e.g., phenolic acids, flavonoids, and derivatives), some were also present in the *P. lusitanica* extracts reported herein (Table 2), likely responsible for the inhibition observed. Hydroethanolic extracts of *P. armeniaca* leaves produced 50% inhibition (IC_50_) of α-amylase at concentrations of 3.32–7.52 mg/mL [55], values well above the highest concentration tested in this study (1 mg/mL; Table 4). In addition, Wojdyło and Nowicka [55] reported, for the same extracts, lower IC_50_ values for α-glucosidase (0.71 to 3.35 mg/mL) inhibition, supporting a higher affinity of the components for α-glucosidase when compared to α-amylase, as observed herein for *P. lusitanica* extracts (Table 4). The low and moderate anti-α-amylase and anti-α-glucosidase activity, respectively, indicates the low antidiabetic effect of these extracts, with a low hypoglycemic potential.

## 3. Materials and Methods

### 3.1. Chemicals and Reagents

Sodium nitrate, aluminum chloride, and sodium hydroxide, all extra pure (>99%), and methanol were acquired from Merck (Merck, Darmstadt, Germany). Folin–Ciocâlteu’s reagent, 3,4,5-trihydroxybenzoic acid (gallic acid) and acetic acid, both extra pure (>99.0%), and sodium hydroxide were purchased from Panreac (Panreac Química S.L.U., Barcelona, Spain). Sodium molybdate (99.5%) was obtained from Chem-Lab (Chem-Lab N.V., Zedelgem, Belgium). The compounds 2,2-azino-bis (3-ethylbenzothiazoline-6-sulfonic acid) diammonium salt (ABTS), 2,2-diphenyl-1-picrylhidrazyl radical (DPPH), potassium phosphate, catechin, potassium persulfate, sodium acetate, 2,4,6-tripyridyl-s-triazine (TPTZ iron reagent), acetic acid, hydrochloric acid, and iron(III) chloride were obtained from Sigma-Aldrich/Merck (Algés, Portugal). Additionally, 6-hydroxy-2,5,7,8-tetramethylchroman-2-carboxylic acid (Trolox) was purchased from Fluka Chemika (Fluka Chemika, Neu-Ulm, Switzerland). The standards for chromatographic determinations were purchased from Sigma-Aldrich/Merck. Methanol, acetonitrile, and acetic acid (LC–MS-grade solvents) were provided by J.T. Baker (Philipsburg, NJ, USA). Milli-Q purified water (Millipore, Bedford, MA, USA) was used for all the extraction and chromatographic analyses. Enzymes and reagents for all enzymatic activities were purchased from Sigma-Aldrich/Merck.

### 3.2. Plant Material

To carry out this study, fruits of *Prunus lusitanica* L. were harvested, fully matured (characterized by a homogeneous purple color along the bunch) in early October, from three different locations: (1) at the University of Trás-os-Montes and Alto Douro (UTAD, Vila Real, Portugal; plants belonging to UTAD’s Botanical Garden collection), (2) in Pardieiros (Arganil, Portugal; voucher HVR 22669), which is currently included in the protected region of Serra do Açor, and (3) in Mata da Margaraça (Serra do Açor, Portugal; voucher HVR22670). These locations are denoted as PL1, PL2 and PL3, respectively. The specimens were properly identified by UTAD’s Botanical Garden office, where the vouchers were created and deposited.

After being collected, the samples were lyophilized (freeze-drier VirTis Benchtop Pro with Omnitronics TM), ground (using a coffee grinder), and stored in a hermetically sealed container protected from light until further analysis.

### 3.3. Preparation of Extracts from Prunus lusitanica L. Fruits

From each sample, three extracts were prepared by mixing 1.2 g of powder (lyophilized and grounded fruits) with 45 mL of hydroethanolic solution (70:30, % *v*/*v*). The mixture was homogenized and placed under stirring in an orbital shaker (GFL 3005, GEMINI, Apeldoorn, The Netherlands), at room temperature, for 30 min.

Afterward, the mixtures were centrifuged at 2291× *g*, 4 °C, for 15 min (Sigma 2-16KL Refrigerated Centrifuges, Sigma Laborzentrifugen, Berlin, Germany) and the supernatants were collected (this process was repeated four times). The resulting supernatants were combined and filtered through 0.2 μm regenerated polyamide membrane filters (NL 16 Whatman^TM^). Ethanol was removed by repeated evaporation under vacuum at 35 °C, and the residue was freeze-dried. Solids were frozen for further analysis.

### 3.4. Determination of the Antioxidant Capacity

The antioxidant potential of fruit extracts was evaluated using three different spectrophotometric methods: ABTS and DPPH radical-scavenging methods, and FRAP (ferric ion-reducing antioxidant power). Procedures for DPPH and ABTS were carried out according to Lemos et al. (2020) [79], whereas the procedure for FRAP was carried out according to Beltrão et al. (2020) [80]. The methodologies were performed using 96-well microplate-adapted assays (PrimeSurface MS-9096MZ, Frilabo, Maia, Portugal) using a microplate reader (Multiskan GO Microplate Photometer, Thermo Fisher Scientific, Vantaa, Finland).

ABTS radicals were produced during a 12–16 h reaction period by mixing 5 mL of ABTS stock solution (7.0 mM in water) with 88 μL of potassium persulfate (148 mM), which was then diluted to a working solution, using sodium acetate buffer (20 mM, pH 4.5), at an absorbance of 0.70 at 734 nm. Then, 188 μL of ABTS working solution and 12 μL of each extract (distilled water served as blank) were combined and allowed to react under a light-protected environment, and the absorbance at 734 nm was measured after 30 min. Results were given in millimoles of Trolox equivalents per gram of dry extract (mmol TE/g dw). DPPH radical (8.87 mM in methanol) was diluted in a 70:30 (% *v*/*v*) methanol/water solution to achieve an absorbance of 1.00 at 520 nm before being used to create the DPPH working solution. Then, 190 μL of DPPH working solution and 10 μL of extract (using 70% hydromethanolic solution as blank) were mixed and incubated for 30 min (protected from light, at room temperature), after which the absorbance at 520 nm was measured to assess radical-scavenging activity. The scavenging capacity of samples was determined using interpolation of the Trolox calibration curve for DPPH and ABTS assays. Results were expressed as millimoles of Trolox equivalents per gram of dry extract (mmol TE/g dw). In order to measure the ferric ion-reducing antioxidant power (FRAP), extracts (20 μL) were added to the microplate, along with 280 μL of the FRAP working solution, consisting of a mixture at 10:1:1 (*v*:*v*:*v*) of acetate buffer (300 mM, pH 3.6), ferric chloride (20 mM in water), and TPTZ (10 mM in hydrochloric acid). This mixture was incubated for 30 min at 37 °C in the dark. Absorbance was then read at 593 nm. Once more, Trolox served as the reference, and the results were given in mmol TE/g dw.

### 3.5. HPLC-PAD–ESI-MS/MS Analysis of the Quantitative (Poly)phenolic Profile of Prunus lusitanica L. Fruit Extracts

The identification and quantification of phenolic compounds were performed according to Abellán et al. (2021) [81], with minor modifications. Briefly, chromatographic separations were carried out on a Kinetex Luna C18 column (250 × 46 mm, 5 µm particle size; Phenomenex, Macclesfield, UK) with a SecurityGuard C18-ODS cartridge system (Phenomenex). The chromatographic resolution of the phenolic profile was achieved using deionized water/formic acid (99:1, % *v*/*v*) (A) and acetonitrile (B) as the chromatographic solvents according to the following gradient (time in min, %B): (0, 5%), (30, 25%), (35, 50%), (37, 50%), and (38, 95%). The flow rate was 1 mL/min, and the injection volumes were 20 µL. The HPLC system was equipped with an Agilent 1100 Series diode array and a mass detector in series (Agilent Technologies, Waldbronn, Germany). It consisted of a G1312A binary pump, a G1313A auto-sampler, a G1322A degasser, and a G1315B photodiode array detector controlled by ChemStation software version 08.03 (Agilent Technologies, Waldbronn, Germany). Spectroscopic data from all peaks were accumulated in the range of 240–600 nm, and the spectral data were recorded at 280 nm, 320 nm, 330 nm, and 520 nm.

The mass detector was a G2445A Ion-Trap Mass Spectrometer equipped with an electrospray ionization (ESI) system and controlled by LCMSD software version 4.1 (Agilent, Waldbronn, Germany). Nitrogen was used as the nebulizing gas, at 60 psi with flow adjusted to 11 L/min. The heated capillary and voltage for ionization were maintained at 350 °C and 5 kV, respectively. Collision-induced fragmentation experiments were performed in the ion trap using helium as collision gas, with voltage ramping cycles from 0.3 up to 2 V. The full scan covered the range from *m*/*z* 100 to *m*/*z* 1600. Mass spectrometry data were acquired in negative and positive ionization modes. Total ion chromatograms were recorded as two alternating automatic scan events: full-scan mass spectra (MS) and MS/MS for fragmentation of the most abundant molecular ions. Identification of individual phenolic compounds was performed on the basis of retention time (min), parent ions, and fragmentation patterns, in comparison with authentic standards or, when not available, according to descriptions available in literature. Phenolic compounds were quantified by PDA chromatograms recorded at the above-indicated wavelengths, using, for each day of analysis, freshly prepared calibration curves.

### 3.6. In Vitro Cell-Based Assays

#### 3.6.1. Cell Culture Maintenance and Handling

In this study, two human cell lines, Caco-2 (human colon adenocarcinoma; Cell Lines Service (CLS), Eppelheim, Germany) and HepG2 (human hepatocellular carcinoma; ATCC^®^ Number: HB-8065TM), as well as a mouse cell line, RAW 264.7 (mouse macrophages, Abelson murine leukemia virus-induced tumor; CLS, Eppelheim, Germany), were used to evaluate the antiproliferative and anti-inflammatory activities of *P. lusitanica* fruit extracts. Cells were cultured in DMEM (Dulbecco’s modified Eagle medium) supplemented with 1 mM L-glutamine, 10% (*v*/*v*) fetal bovine serum (FBS), 100 U/mL penicillin, and 100 µg/mL streptomycin (all reagents from Gibco, Alfagene, Lisbon, Portugal), and then kept in an incubator (37 °C, 5% CO_2_/95% air, controlled humidity). Cells were treated and handled as reported by Silva et al. (2020) [82]. Briefly, near-confluence Caco-2 and HepG2 cells were treated with trypsin-EDTA, and the reaction was stopped using complete culture medium (1:1, trypsin/culture medium); in the case of RAW 264.7 cells, the cells were scratched off the flasks using a cell scratcher (Orange Scientific; Braine-L’Alleud, Belgium). Then, cells were counted, diluted in fresh culture medium at a final density of 5 × 10^4^ cells/mL, and seeded in 96-well culture microplates.

#### 3.6.2. Cell Viability/Cytotoxicity Assay

In order to determine the effect of *P. lusitanica* fruit extracts on cell viability, Caco-2, HepG2, and RAW 264.7 cells seeded in 96-well microplates (see Section 3.6.1.) were allowed to adhere for 24 h before being subjected to extracts. After this period of time, the culture medium was removed, being immediately replaced with the test solutions replaced (extracts diluted in FBS-free culture medium at different concentrations: 25, 50, 100, 200, 500, and 750 µg/mL). Cells were then incubated for 24 or 48 h (in the incubator). Cell viability was assessed using the Alamar Blue (AB) metabolic indicator. After cell incubation with the extracts, the test solutions were removed, and the cells were exposed to 100 µL/well of AB solution (at 10% (*v*/*v*), in FBS-free culture medium). Next, 4–5 h after exposure to AB, the absorbance was read at 570 nm and 620 nm using a microplate reader (Multiskan EX; MTX Lab Systems, Inc., Bradenton, Fl, USA). Results were expressed as the cell viability (% of control; i.e., of positive control or nontreated cells), calculated as described by Andreani et al. (2014) [83].

#### 3.6.3. Assessment of Anti-Inflammatory Activity

To assess the anti-inflammatory activity of *P. lusitanica* fruit extracts, the RAW 264.7 cell model stimulated by lipopolysaccharide (LPS) was used. RAW 264.7 cells were handled as described previously [82]. Briefly, RAW 264.7 cells seeded in 96-well plates (5 × 10^4^ cells/well) were allowed to adhere and stabilize for 48 h, before being exposed to noncytotoxic concentrations (25–100 µg/mL) of *P. lusitanica* fruit extracts for 24 h. Noncytotoxic concentrations were determined on the basis of the cell viability assay. Then, the extract solutions were removed, and the cells were gently rinsed with sterile phosphate buffer (PBS) to remove all traces of extracts (to avoid interference with the Griess assay, due to the chromophore effect), before being exposed to FBS-free culture medium (nonexposed cells, negative controls) or LPS (at 1 µg/mL in FBS-free culture medium) solution for 24 h (maintained in incubator). LPS induces the production of nitric oxide (NO) by RAW 264.7 cells, which is released into the incubation medium. After 24 h incubation, 50 µL of supernatant was removed from each well and transferred into a new 96-well plate. Then, 50 µL of Griess reagent [1% (*w*/*v*) sulfanilamide prepared in 5% (*w*/*v*) H_3_PO_4_ (*v*/*v*) and 0.1% (*w*/*v*) *N*-(1-naphthyl) ethylenediamine dihydrochloride in water] was added to each well. After 15 min incubation (room temperature, in the dark), the absorbance was read at 550 nm. The quantification of released NO was performed using a calibration curve obtained with sodium nitrite (NaNO_2_; range: 0 to 100 µM). Results were expressed as a percentage of the positive control (i.e., nitrite production by cells that were not exposed to extracts but stimulated with LPS) set to 100%, i.e., 0% anti-inflammatory effect.

### 3.7. In Vitro Bioassay for the Inhibition of Enzymes Involved in Several Relevant Metabolic Processes

The below-described enzyme inhibition assays were carried out in 96-well plates, and the absorbance of the respective products was read using a microplate reader.

#### 3.7.1. Acetylcholinesterase (AChE) Inhibition Assay

The AChE inhibition assay was performed according to Elman’s method [84], with some modifications as described by Martins-Gomes et al. (2022) [85]. Briefly, to the extract (50 µL; at 0.5 or 1 mg/mL), DTNB (125 µL; 0.3 mM, in 50 mM Tris-HCl, pH 8) and acetylthiocholine iodide (ATCI; 25 µL; 1.5 mM). AChE (25 µL; 0.026 U/mL, in 20 mM Tris-HCl, pH 7.5) were sequentially added after 2 min of incubation. Then, the mixture was left to incubate for 10 min at 25 °C, after which the absorbance (Abs) was measured at 405 nm. Equation (1) was used to calculate the inhibition (%).

Inhibition (%) = [(Abs control − Abs sample)/(Abs control)] × 100.(1)

#### 3.7.2. Tyrosinase Inhibition Assay

The tyrosinase inhibition assay was performed on the basis of L-DOPA enzymatic oxidation, as described in [85]. Briefly, L-DOPA (40 µL; 2.5 mM) and phosphate buffer (80 µL; 50 mM, pH 6.8) were added to each extract (25 µL; at 0.5 or 1 mg/mL) and allowed to incubate for 2 min at 37 °C. Then, 40 µL of tyrosinase (40 U/mL, in phosphate buffer (50 mM, pH 6.5)) was added to start the reaction. Absorbance was measured at 492 nm after 10 min incubation at 37 °C. Kojic acid (1 mg/mL, in water) was used as the positive control, and the inhibition (%) was calculated using Equation (1).

#### 3.7.3. Elastase Inhibition Assay

The anti-elastase activity of *P. lusitanica* fruit extracts was evaluated following a previously described procedure [21,62,86]. Briefly, to 50 µL of each extract (at 0.5 or 1 mg/mL), 160 µL of Tris-HCl buffer (0.2 M, pH 8) and 20 µL of *N*-(methoxysuccinyl)-ala-ala-pro-val-4-nitroanilide (0.8 mM, in buffer) were added. Then, this mixture was incubated for 10 min at room temperature. Next, elastase solution (20 µL; 0.4 U/mL in Tris-HCl buffer) was added to initiate the reaction. The absorbance was measured at 410 nm, after 20 min of incubation. Equation (1) was used to calculate the inhibition (%).

#### 3.7.4. α-Amylase Inhibition Assay

For the α-amylase inhibition assay, the method described by Torres-Naranjo et al. (2016) [87] was modified as described in [21]. A mixture of each extract (5 µL; 0.5 or 1 mg/mL), PBS (35 µL), and starch solution (35 µL; 0.05%, pH 7) was preincubated for 2 min at 37 °C. Then, α-amylase solution (20 µL; 50 µg/mL; in phosphate buffer: 10 mM, pH 6.9) was added, and the mixture was allowed to react for 10 min at 37 °C. After incubation, the reaction was stopped by adding 50 µL of 0.1 M HCl solution. Then, 150 µL of Lugol solution (0.5 mM I_2_, 0.5 mM KI, in water) was added, and the mixture was incubated for 15 min. Absorbance was measured at 580 nm. Acarbose (1 mg/mL) was used as the positive control. Inhibition (%) was calculated according to Torres-Naranjo et al. (2016) [87].

#### 3.7.5. α-Glucosidase Inhibition Assay

The α-Glucosidase inhibition was performed using rat intestinal acetone powder (Sigma-Aldrich/Merck) as a crude enzyme extract, as described previously [21]. Briefly, crude enzyme extract (250 mg) diluted in sodium phosphate buffer (10 mL; 0.1 M, pH 7.0) was centrifuged (5000× *g*, 20 min, 4 °C), and the supernatant was collected. To 50 µL of each extract (at 0.5 or 1 mg/mL), 100 µL of supernatant was added, and the mixture was incubated for 10 min at room temperature. Then, 50 µL of *p*-nitrophenyl-α-D-glucopyranoside (5 mM, in sodium phosphate buffer) were added, and the mixture was further incubated (30 min, 37 °C). After incubation, the absorbance was measured at 405 nm. Acarbose (1 mg/mL) was used as the positive control. Inhibition (%) was calculated according to Equation (1).

### 3.8. Data and Statistical Analysis

All assays were carried out in triplicate (n = 3), and the results were expressed as the mean ± standard deviation (SD). For the cytotoxicity assays, the IC_50_ (the concentration that reduces cell viability by 50%) values were calculated as described by Silva et al. (2019) [44]. The statistical differences were determined through analysis of variance (ANOVA) and a multiple range test (Tukey’s test) at *p* < 0.05. Statistical analyses and graphic design were performed using GraphPad Prism (Version 8; GraphPad Software Inc., San Diego, CA, USA).

## 4. Conclusions

In conclusion, the phytochemical profile of *P. lusitanica* fruit HE extracts was identical regarding the types of phytochemicals, independently of the location of harvest (PL1, PL2, and PL3); however, differences in the quantities of particular compounds were observed, potentially reflecting the edaphoclimatic differences across locations. Extracts from *P. lusitanica* fruits collected in Vila Real (PL1) had higher amounts of total phenolic compounds and total hydroxycinnamic acids, but contained a lower amount of total flavan-3-ols. Samples from Mata da Margaraça (PL3 extracts) contained higher amounts of total flavan-3-ols (more than twice that of PL1 sample) and total anthocyanins (more than twice that of Pardieiros sample, PL2). *P. lusitanica* fruit extracts generally exerted a low cytotoxic/antiproliferative effect; however, in HepG2 cells, a dose- and time-dependent decrease in cell viability was observed for concentrations higher than 100 μg/mL. The extracts showed high anti-inflammatory capacity, with all extracts reducing LPs-induced NO release by more than 50% at 100 μg/mL. The extracts also exhibited high neuroprotective potential and moderate antiaging and antidiabetic effects. Given that these fruits are not currently used for human consumption, it would be interesting in the future to assess whether they contain any antinutrients or potentially toxic compounds for humans, in order to promote these fruits as functional foods or as ingredients for the food industry. Additionally, the compounds present in these extracts are worth exploring for the development of new drugs of interest to the pharmaceutical and cosmetic industries.

## Figures and Tables

**Table 1:** Extraction yields (%, *w*/*w*) and in vitro antioxidant capacity of *P. lusitanica* L. fruit hydroethanolic extracts.

	Extraction Yield (%, *w*/*w*)	Antioxidant Activity (mmol TE/g dw)		
		FRAP	DPPH	ABTS
PL1	55.28 ± 1.53 ^ab^	0.36 ± 0.01 ^a^	0.36 ± 0.01 ^b^	0.45 ± 0.01 ^a^
PL2	59.70 ± 1.12 ^b^	0.33 ± 0.02 ^a^	0.32 ± 0.01 ^a^	0.42 ± 0.02 ^a^
PL3	54.05 ± 3.12 ^a^	0.43 ± 0.01 ^b^	0.40 ± 0.01 ^c^	0.52 ± 0.02 ^b^

Note: Results are presented as the mean ± SD (n = 3); mmol Trolox eq/g, mmol of Trolox equivalents per gram of dry extract. PL1, PL2, and PL3 refer to the three different locations where *P. lusitanica* fruits were harvested (see Section 3 for details). In the same column, different letters denote significant differences in extracts across locations according to Tukey’s test (*p* < 0.05).

**Table 2:** Quantitative (poly)phenolic profile (mg/g dw) of *Prunus lusitanica* L. fruit hydroethanolic extracts from three different locations.

	R.T. (min)	λ Max (nm)	[M − H]^−^/[M + H]^+^ (*m/z*)	MS ^n^ [M – H]^−^/[M + H]^+^ (*m/z*)	Identification	Location		
						PL1	PL2	PL3
***Hydroxycinnamic acids***								
**1**	14.4	324	353/-	**191**,179/-	3-*O*-Caffeoylquinic acid	0.19 ± 0.01 ^b^	0.15 ± 0.01 ^a^	0.13 ± 0.00 ^a^
**2**	15.2	328	503/-	341,323,179,**161**,143,135/-	Caffeoyl di-hexoside	0.67 ± 0.03 ^c^	0.32 ± 0.02 ^b^	0.15 ± 0.01 ^a^
**3**	17.3	306	487/-	341,307,173,163,**145**/-	*p*-Coumaroyl-3-*O*-sucrose	1.55 ± 0.09 ^a^	1.74 ± 0.14 ^a^	2.26 ± 0.23 ^b^
**4**	18.0	310	337/-	191,**163**, 119/-	3-*p*-Coumaroylquinic acid	1.55 ± 0.14 ^c^	1.16 ± 0.04 ^b^	0.61 ± 0.07 ^a^
**5**	18.9	312	487/-	341,307,179,163,**145**/-	Caffeic acid-*O*-(coumaroyl)hexoside	4.82 ± 0.41 ^c^	3.35 ± 0.12 ^b^	1.64 ± 0.20 ^a^
**7**	21.9	326	353/-	191,179,**173**,155,135,127,111/-	Caffeoyl-isocitrate	1.70 ± 0.19 ^b^	2.03 ± 0.25 ^a^	2.36 ± 0.12 ^a^
**10**	26.4	312	337/-	**173**,163,155,137,127/-	4-*p*-Coumaroylquinic acid	6.28 ± 0.47 ^c^	3.77 ± 0.38 ^b^	2.16 ± 0.24 ^a^
**11**	27.0	312	529/-	**487**,349,307,173,162,145/-	Mono-*O*-acetyl-3-*O*-*p*-coumaroylsucrose isomer	17.97 ± 1.27 ^a^	19.96 ± 1.82 ^a^	27.24 ± 2.71 ^b^
**12**	27.4	314	529/-	**487**,349,307,173,162,145/-	Mono-*O*-acetyl-3-*O*-*p*-coumaroylsucrose isomer	2.35 ± 0.30 ^b^	2.01 ± 0.14 ^b^	1.26 ± 0.04 ^a^
**13**	32.4	314	571/-	**529**,511,487,469,393,383,341,307/-	Di-*O*-acetyl-3-*O*-*p*-coumaroyl sucrose isomer	20.15 ± 1.50 ^b^	17.85 ± 0.95 ^b^	13.93 ± 1.03 ^a^
**14**	32.7	308	571/-	**529**,511,487,469,425,383,367,349,341,307,217,173,171,163/-	Di-*O*-acetyl-3-*O*-*p*-coumaroyl sucrose isomer	1.51 ± 0.20 ^b^	0.87 ± 0.06 ^a^	0.74 ± 0.01 ^a^
**15**	33.2	314	571/-	**529**,511,487,469,425,383,367,341,307,289/-	Di-*O*-acetyl-3-*O*-*p*-coumaroyl sucrose isomer	1.52 ± 0.11 ^b^	1.41 ± 0.06 ^b^	1.05 ± 0.08 ^a^
**16**	33.8	314	613/-	**571**,559,553,451,449,425,407,289,273/-	Tri-*O*-acetyl-3-*O*-*p*-coumaroyl sucrose isomer	1.05 ± 0.12 ^b^	0.76 ± 0.11 ^a^	0.59 ± 0.05 ^a^
**17**	34.9	308	613/-	**571**,553,529,511,425,383,349,289,217,163/-	Tri-*O*-acetyl-3-*O*-*p*-coumaroyl sucrose isomer	2.54 ± 0.20 ^b^	1.26 ± 0.01 ^a^	1.37 ± 0.15 ^a^
**18**	35.9	306	571/-	529,**511**,487,469,451,422,349,331,307,289,271,259,231,214,173,145/-	Di-*O*-acetyl-3-*O*-*p*-coumaroyl sucrose isomer	1.23 ± 0.11 ^a^	1.14 ± 0.11 ^a^	1.15 ± 0.16 ^a^
**19**	36.2	310	613/-	**571**,553,529,511,487,469,467,425,407,383,349,307,277,228,219,201,163/-	Tri-*O*-acetyl-3-*O*-*p*-coumaroyl sucrose isomer	19.65 ± 1.25 ^b^	16.78 ± 0.66 ^a^	14.64 ± 1.25 ^a^
**22**	37.6	316	613/-	571,**553**,511,493,469,425,365,349,331,307,289,271,269,245,214,187,163/-	Tri-*O*-acetyl-3-*O*-*p*-coumaroyl sucrose isomer	6.39 ± 0.63 ^b^	5.19 ± 0.01 ^a^	5.69 ± 0.30 ^ab^
**23**	38.1	316	613/-	571,**553**,511,493,469,451,425,407,391,331,303,287,271,214,197/-	Tri-*O*-acetyl-3-*O*-*p*-coumaroyl sucrose isomer	3.39 ± 0.33 ^b^	2.39 ± 0.14 ^a^	3.27 ± 0.36 ^b^
**24**	38.2	318	613/-	571,**553**,511,493,451,407,391,389,349,331,318,245,163/-	Tri-*O*-acetyl-3-*O*-*p*-coumaroyl sucrose isomer	5.78 ± 0.51 ^b^	4.38 ± 0.38 ^a^	4.86 ± 0.31 ^ab^
**25**	38.6	316	655/-	613,**595**,553,535,493,391,349,330,313,287,270/-	Tetra-*O*-acetyl-3-*O*-*p*-coumaroylsucrose isomer	6.81 ± 0.41 ^b^	3.66 ± 0.07 ^a^	3.33 ± 0.28 ^a^
**26**	39.3	39398, sh334	655/-	613,**595**,553,535,511,493,393,331/-	Tetra-*O*-acetyl-3-*O*-*p*-coumaroylsucrose isomer	25.53 ± 0.49 ^b^	22.17 ± 0.17 ^a^	23.64 ± 1.19 ^a^
***Secoiridoids***								
**6**	21.6	328	581/-	**545**,503,341,323,235,161/-	6-*O*-*β*-*D*-Glucosyl swertiamarin (tentative)	1.70 ± 0.19 ^b^	1.46 ± 0.13 ^ab^	1.25 ± 0.03 ^a^
***Flavan-3-ols***								
**8**	25.3	278	289/-	**245**,205,203,179,165/-	Catechin	1.19 ± 0.15 ^a^	2.93 ± 0.19 ^c^	2.48 ± 0.09 ^b^
**9**	25.9	280	865/-	739,713,**695**,577,575,451,407,363,289,287,173,163/-	B-type proanthocyanidin trimer	4.73 ± 0.05^a^	8.68 ± 0.58 ^b^	10.92 ± 0.81 ^c^
***Flavonols***								
**20**	36.9	340	463/-	**301**,271,255,229,213,193,179,151,121/-	Quercetin-3-*O*-glucoside	0.18 ± 0.01 ^b^	0.11 ± 0.01 ^a^	0.20 ± 0.02 ^b^
**21**	37.1	356	609/-	**301**,271,255,229,211,193,179,151,121,107/-	Quercetin-3-*O*-rutinoside	2.40 ± 0.19 ^b^	1.19 ± 0.11 ^a^	2.33 ± 0.08 ^b^
***Anthocyanins***								
**27**	28.6	518	-/449	-/366,307,**287**,227,213,203,187,160	Cyanidin-3-*O*-glucoside	2.00 ± 0.01 ^b^	0.67 ± 0.01 ^a^	2.90 ± 0.06 ^c^
**28**	30.4	520	-/595	-/467,329,**287**,269,259,252,219,127	Cyanidin-3-(6-*trans*-*p*-coumaroyl)glucoside	17.08 ± 0.28 ^b^	10.01 ± 0.27 ^a^	20.94 ± 2.21 ^c^
					**Total phenolic compounds**	162.29 ± 4.55 ^b^	137.38 ±1.76 ^a^	153.11± 8.58 ^b^
					**Total hydroxycinnamic acids**	133.02 ± 4.38 ^b^	112.34 ± 1.34 ^a^	112.07 ± 5.47 ^a^
					**Total secoiridoids**	1.70 ± 0.19 ^b^	1.46 ± 0.13 ^ab^	1.25 ± 0.03 ^a^
					**Total Flavan-3-*ols***	5.92 ± 0.16 ^a^	11.61 ± 0.42 ^b^	13.41 ± 0.77 ^c^
					**Total Flavonols**	2.58 ± 0.19 ^b^	1.30 ± 0.11 ^a^	2.53 ± 0.10 ^b^
					**Total Anthocyanins**	19.08 ± 0.29 ^b^	10.68 ± 0.28 ^a^	23.85 ± 2.27 ^c^

Note: Data are shown as the mean ± SD (n = 3). Different letters within the same row indicate significant differences in concentrations of extracts from the fruits retrieved in the different locations, according to the analysis of variance (ANOVA) and the multiple range Tukey test at *p* < 0.05. Fragments identified in bold correspond to the base peak. R.T.—retention time.

**Table 3:** Half-maximal inhibitory concentration (IC_50_; in µg/mL) of *P. lusitanica* extracts for the tested cell lines exposed for 24 and 48 h (see Section 3 for details).

		IC_50_ (µg/mL)		
		Caco-2	HepG2	RAW 264.7
PL1	24 h	-	584.10 ± 25.51 ^c^	-
	48 h	-	352.60 ± 10.00 ^a^	745.20 ± 36.01
PL2	24 h	-	-	-
	48 h	-	501.10 ± 13.45 ^b^	-
PL3	24 h	-	701.30 ± 26.42 ^d^	-
	48 h	-	517.30 ± 22.66 ^b^	-

Note: Significant differences (*p* < 0.05) between samples within the same column are denoted by different letters.

**Table 4:** In vitro assessment of *Prunus lusitanica* L. fruits extracts’ inhibitory activity against acetylcholinesterase (AChE), tyrosinase, elastase, α-glucosidase, and α-amylase.

		Enzymatic Inhibition (% of the Control)				
Extract	Concentration	AChE	Tyrosinase	Elastase	α-Amylase	α-Glucosidase
PL1	0.5 mg/mL	28.33 ± 1.04 ^c^	2.92 ± 0.79 ^a^	n.d.	1.81 ± 0.16 ^a^	14.59 ± 1.92 ^c^
	1 mg/mL	39.41 ± 4.18 ^d^	15.33 ± 3.38 ^c^	n.d.	2.58 ± 0.29 ^b^	15.10 ± 0.88 ^c^
PL2	0.5 mg/mL	23.15 ± 1.04 ^b^	2.00 ± 0.17 ^a^	n.d.	1.55 ± 0.92 ^ab^	12.67 ± 1.28 ^bc^
	1 mg/mL	37.19 ± 0.85 ^d^	11.67 ± 3.6 ^bc^	n.d.	2.03 ± 0.66 ^ab^	15.21 ± 0.96 ^c^
PL3	0.5 mg/mL	17.98 ± 1.04 ^a^	4.75 ± 1.80 ^a^	n.d.	1.31 ± 0.39 ^a^	1.98 ± 2.40 ^a^
	1 mg/mL	34.24 ± 3.13 ^d^	9.48 ± 0.06 ^b^	n.d.	2.14 ± 0.47 ^ab^	10.63 ± 0.80 ^b^

Notes: Results are presented as the mean ± SD (n = 3); n.d.: not detected. In the same column, different letters denote significant differences across locations according to Tukey’s test (*p* < 0.05).

## Data Availability

Not applicable.
